# Supplementary material for: Exploring the Antidiabetic Properties of Polyalthia longifolia Leaf and Stem Extracts: In Vitro α-Glucosidase and Glycation Inhibition
Source: Molecules. 2025 Oct 31;30(21):4264. doi: 10.3390/molecules30214264 (PMC12609144; doi:10.3390/molecules30214264)
Supplement: Supplementary file 1 [file molecules-30-04264-s001.zip › molecules-3930996-supplementary.pdf]

molecules

MDPI

Supplementary materials

Exploring the Antidiabetic Properties of *Polyalthia longifolia* Leaf and Stem Extracts: In Vitro α-Glucosidase and Glycation Inhibition

Guglielmina Froldi [1,*], Marguerite Kamdem Simo [2,3], Laura Tomasi [1], Giulia Tadiotto [1], Francine Medjiofack Djeujo [1], Xavier Gabriel Fopokam [2], Emmanuel Souana [2], Modeste Lambert Sameza [4], Pierre Michel Jazet [4] and Fabrice Fekam Boyom [2,5]

[1] Department of Pharmaceutical and Pharmacological Sciences, University of Padova, 35131 Padova, Italy; laura.tomasi@virgilio.com (L.T.); giuliatadiotto@gmail.com (G.T.); francine.medjiofackdjeujo@phd.unipd.it (F.M.D.)
[2] Antimicrobial & Biocontrol Agents Unit, Department of Biochemistry, University of Yaoundé I, Yaoundé P.O. Box 812, Cameroon; simomagui@yahoo.fr (M.K.S.); fopokam@gmail.com (X.G.F.); souana.emmanuel@facsciences-uy1.cm (E.S.); fabrice.boyom@fulbrightmail.org (F.F.B.)
[3] Department of Biological Sciences, University of Maroua, Maroua P.O. Box 814, Cameroon
[4] Department of Biochemistry, University of Douala, P O Box 24 157, Douala Cameroon; samezamste@yahoo.com (M.L.S.); mjazet@yahoo.com (P.M.J.)
[5] Advanced Research & Health Innovation Hub, Yaoundé, University of Yaoundé I, Cameroon

* Correspondence: g.froldi@unipd.it; Tel.: + 39-049-827-5092

Abstract

Polyalthia longifolia, a member of the Annonaceae family, is traditionally used for its medicinal properties, including as an antidiabetic remedy, primarily in Asia and sub-Saharan Africa. This study investigated the potential of six *P. longifolia* extracts in counteracting hyperglycemia and diabetes-related complications. Aqueous, ethanol, and methanol extracts from leaves and stems were evaluated for their antihyperglycemic, antiglycation, and antiradical properties using α-glucosidase, BSA, and ORAC assays, respectively. Phytochemical characterization was conducted using TPC and TFC assays, and HPLC analysis identified specific bioactive compounds, including various phenolic compounds (gallic acid, (+)-catechin, epicatechin, caffeic acid, ellagic acid and rosmarinic acid) and flavonoids (luteolin, kaempferol and baicalein). The MTT assay on the human cell line HT-29 assessed the activity of extracts on cell viability, showing slight cytotoxicity. Results demonstrated significant antidiabetic activity of the ethanol and methanol extracts from *P. longifolia* leaves. This study provides new insights into the potential use of *P. longifolia* in diabetes mellitus and supports the valorization of traditional medicinal plants.

Keywords: α-Glucosidase inhibition; polyphenols; flavonoids; AGEs; traditional medicine; Annonaceae; ROS scavenging; HPLC-DAD analysis; antidiabetic activity

Results

Polyalthia longifolia (Sonn.) Thwaites *var. pendula (Annonaceae).*

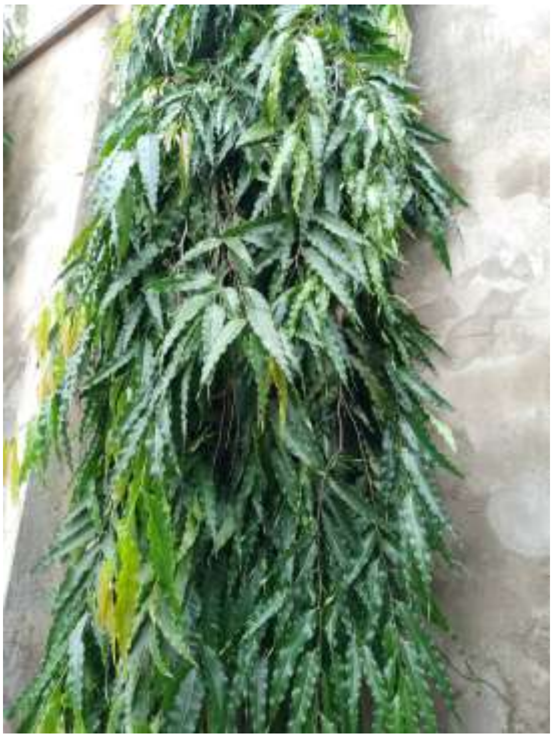

Figure S1 *Polyalthia longifolia* (Sonn.) Thwaites var. p*endula* (Annonaceae).

- **BSA glycation**

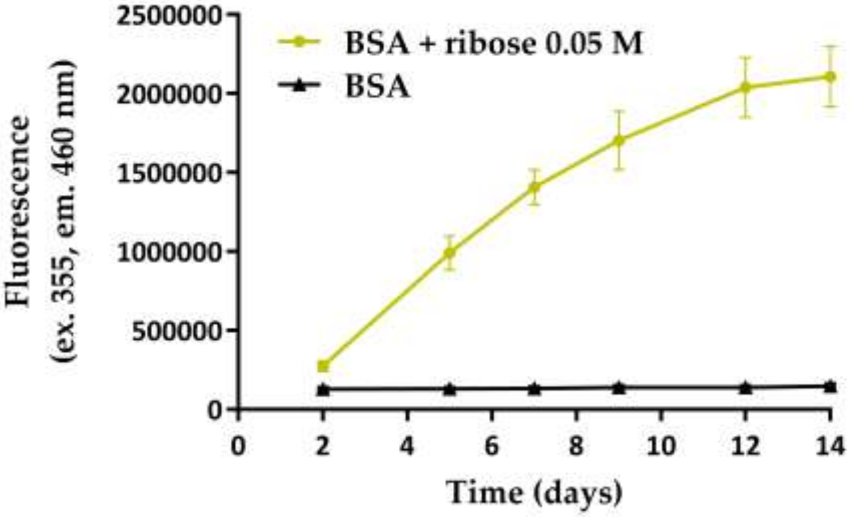

Figure S2 Kinetics of ribose-induced Advanced Glycation End-products (AGEs) formation. Bovine Serum Albumin (BSA) was used as the protein substrate and incubated with ribose at 37 °C for 14 days. AGE formation was monitored by fluorescence spectroscopy. Data points represent mean ± SEM of at least 3 independent experiments, each performed in triplicate. For detailed experimental conditions, see Methods section.

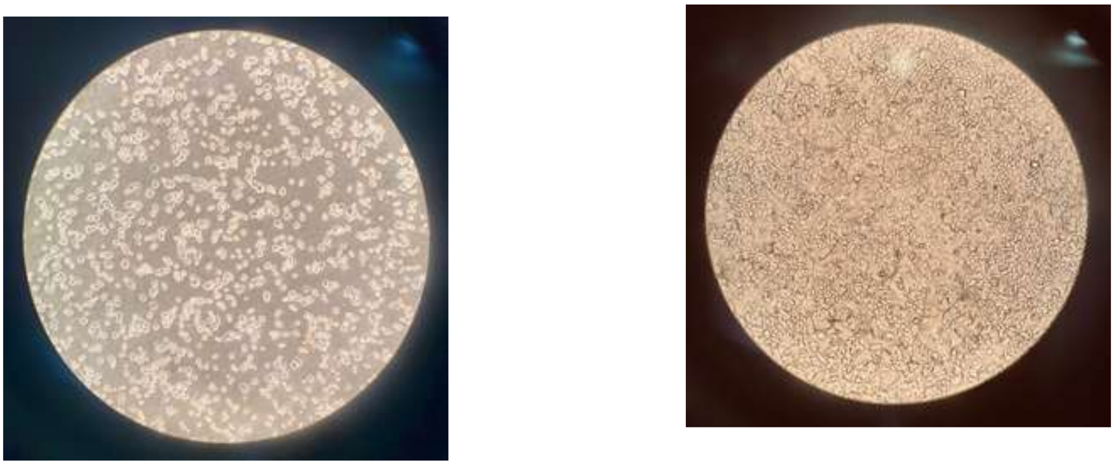

Figure S3 HT-29 human colon adenocarcinoma cells in culture. A: Cells at 48 hours post-seeding in Petri dish. B: Cells at 96 hours post-seeding in Petri dish. Micrographs captured using a NikonTM phase-contrast microscope. Scale bar: 100 μm.

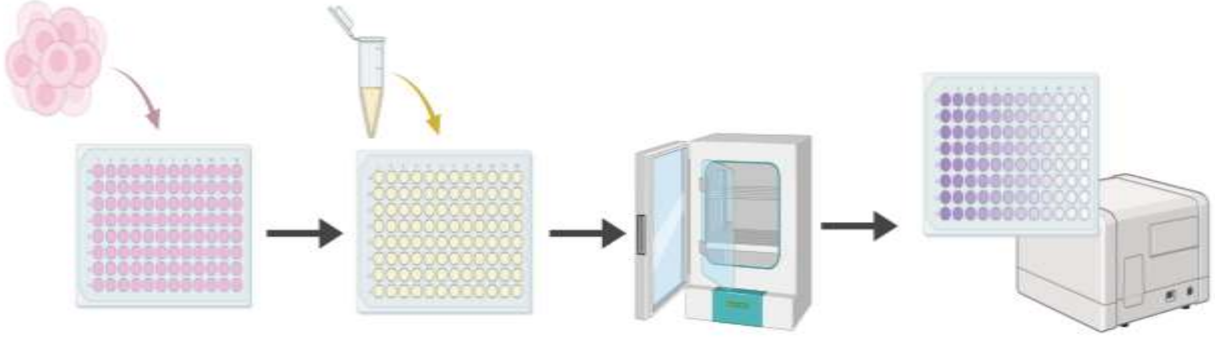

Figure S4 HT-29 cell viability assay protocol. Day 1: Cell seeding (5000 cells/well). Day 2: Treatment with *Polyalthia longifolia* extracts (1-250 μg/mL) and controls (medium only). Day 3: MTT addition, formazan crystal formation and solubilization, absorbance measurement at 570 nm (PerkinElmer Victor Nivo™). Image created using https://www.biorender.com.
